# GSH/ROS Dual-Responsive Supramolecular Nanoparticles Based on Pillar[6]arene and Betulinic Acid Prodrug for Chemo–Chemodynamic Combination Therapy

**DOI:** 10.3390/molecules26195900

**Published:** 2021-09-29

**Authors:** Peng Zhu, Weidan Luo, Jianqiang Qian, Chi Meng, Wenpei Shan, Zhongyuan Xu, Wei Zhang, Xin Liu, Yong Ling

**Affiliations:** 1 State Key Laboratory of Quality Research in Chinese Medicine, Macau Institute for Applied Research in Medicine and Health, Macau University of Science and Technology, Taipa, Macau 999078, China; zp1216834231@gmail.com (P.Z.); luoweidan111@163.com (W.L.); wzhang@must.edu.mo (W.Z.); 2 School of Pharmacy and Jiangsu Province Key Laboratory for Inflammation and Molecular Drug Target, Nantong University, Nantong 226001, China; qiq1273752353@163.com (J.Q.); jsxzmc123456@163.com (C.M.); swp1902@163.com (W.S.); xzy424052148@163.com (Z.X.)

**Keywords:** chemodynamic therapy (CDT), tumor microenvironment (TME), dual-responsive, betulinic acid (BA), pillar[6]arene, glucose oxidase (GOx)

## Abstract

Chemodynamic therapy (CDT) based on intracellular Fenton reactions is attracting increasing interest in cancer treatment. A simple and novel method to regulate the tumor microenvironment for improved CDT with satisfactory effectiveness is urgently needed. Therefore, glutathione (GSH)/ROS (reactive oxygen species) dual-responsive supramolecular nanoparticles (GOx@BNPs) for chemo–chemodynamic combination therapy were constructed via host–guest complexation between water-soluble pillar[6]arene and the ferrocene-modified natural anticancer product betulinic acid (BA) prodrug, followed by encapsulation of glucose oxidase (GOx) in the nanoparticles. The novel supramolecular nanoparticles could be activated by the overexpressed GSH and ROS in the tumor microenvironment (TME), not only accelerating the dissociation of nanoparticles—and, thus, improving the BA recovery and release capability in tumors—but also showing the high-efficiency conversion of glucose into hydroxyl radicals (·OH) in succession through intracellular Fenton reactions. Investigation of antitumor activity and mechanisms revealed that the dramatic suppression of cancer cell growth induced by GOx@BNPs was derived from the elevation of ROS, decrease in ATP and mitochondrial transmembrane potential (MTP) and, finally, cell apoptosis. This work presents a novel method for the regulation of the tumor microenvironment for improved CDT, and the preparation of novel GSH/ROS dual-responsive supramolecular nanoparticles, which could exert significant cytotoxicity against cancer cells through the synergistic interaction of chemodynamic therapy, starvation therapy, and chemotherapy (CDT/ST/CT).

## 1. Introduction

Cancer is a devastating disease with an ever-increasing mortality rate every year, and represents one of the biggest challenges to human health all over the world [1,2]. Chemotherapeutic drugs are still the most important way to treat cancer [3]. Natural-product-based chemotherapeutic drugs remain the most effective way to treat cancer [4]. Of the 175 small molecules approved for cancer treatment from the 1940s to the end of 2014, 75% are of non-synthetic origin, with 49% being either natural products or directly derived therefrom [5]. Betulinic acid (BA) is a natural lupane-type pentacyclic triterpenoid saponin usually isolated from birch tree bark, and is also present in many other botanical sources, demonstrating multiple bioactivities—particularly an antitumor effect via the elevation of ROS (reactive oxygen species) and induction of apoptosis in cancer cells [6,7,8,9,10,11]. However, the applications of BA are limited due to its poor water solubility, modest therapeutic effectiveness, poor bioavailability, inappropriate tissue distribution, and short plasma half-life [12]. Recently, various BA-based nanoformulations have been designed and developed to improve its oral bioavailability and enhance its therapeutic effectiveness [13,14,15]; however, most of them are not responsive to specific tumor microenvironments and, thus, are not sufficiently selective to exert potent cytotoxicity against tumor cells.

Chemodynamic therapy (CDT) has received great attention since the Fenton reaction was first employed to elevate intracellular oxidative stress by converting intratumoral or intracellular hydrogen peroxide (H_2_O_2_) into hydroxyl radicals (·OH) to kill cancer cells [16,17,18]. The tumor microenvironment (TME) involves acidity (pH ~6.0–6.5) [19] and the overproduction of H_2_O_2_ (~50–100 μM) due to mitochondrial dysfunction [20], which can activate the Fenton reaction. Therefore, the short half-life and high oxidation ability of ·OH induced via Fenton reaction only generates and performs the effective damage at the tumor site, avoiding side effects to surrounding tissues [21]. However, the mild acidity of the tumor microenvironment is not the ideal pH for the Fenton reaction (optimal pH is 2~4) [10]. Moreover, the overexpressed H_2_O_2_ in the TME is not sufficient to produce the high levels of ·OH in succession for cancer treatments. Glucose oxidase (GOx) can selectively catalyze the oxidation of glucose into gluconic acid and H_2_O_2_ with high efficiency [22,23], which is the perfect characteristic for improving acidity and H_2_O_2_ levels in tumors and, thus, improving the CDT via the Fenton reaction [24]. Moreover, cancer cells express an exceeding desire for glucose to supply energy for proliferation, and are more sensitive to changes in glucose concentration than normal cells, depending on the Warburg effect [25,26]. Thus, the depletion of intratumoral glucose by GOx can effectively consume the energy for tumor growth and starve them to death, while also improving the effects of CDT [27].

Supramolecular nanoparticles constructed via non-covalent interactions have attracted widespread attention in the field of drug delivery, due to their simple synthesis procedure, reversibility, and switchable structures [28]. Moreover, they are sensitive to stimuli, with notable responsiveness [29,30]. Pillararenes, the fifth generation of supramolecule, can serve as hosts and bind with the specific functional group of various guests via hydrogen bonding, covalent bonding, π–π stacking interaction, electrostatic interaction, and ionic interaction [31,32,33,34], showing better stability and bioactivity [35,36,37,38,39,40].

**Scheme 1:**
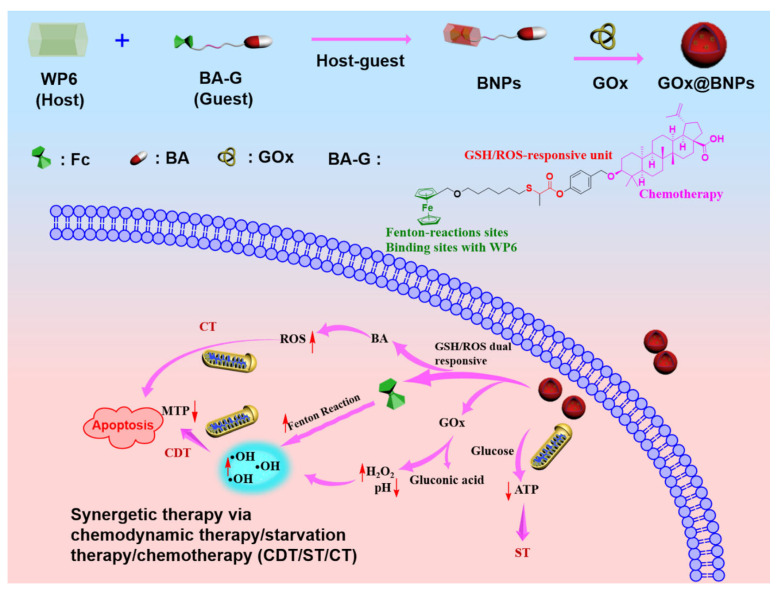
Schematic illustration of the preparation of supramolecular nanoparticles and their GSH/ROS dual-responsive drug release and anti-proliferative mechanisms against A549 cancer cells.

Therefore, the tumor microenvironment (high GSH and ROS)-responsive supramolecular nanoparticles were designed and constructed via the host–guest interactions of water soluble pillar[6]arene with the ferrocene-modified anticancer product BA prodrug (BA-G). Thioether has been employed to bridge ferrocene and the natural anticancer product BA, which can be highly effectively triggered by both the high GSH and ROS in the tumor microenvironment for drug delivery [41]. Then, the encapsulation of GOx in the nanoparticles not only depletes glucose to starve cancer cells, but also improves acidity and H_2_O_2_ levels in tumors, thus improving the CDT to produce hydroxyl radicals with high oxidation ability for killing cancer cells. The thioether-bridged BA prodrug could be a response to the TME, and the release of BA can exert chemotherapy via the elevation of ROS in cancer cells. The novel supramolecular nanoparticles can be tailored to the specific tumor microenvironment, not only improving the efficacy of tumor inhibition while reducing biological risks and the side effects of BA, but also exerting the multimodal synergistic treatment of chemodynamic/starvation/chemotherapy against cancer cells (Scheme 1).

In this research, WP6 could induce the self-assembly of the nanoparticles via host–guest interactions between the ferrocene moiety of BA-G and WP6. Ferrocene is the binding site. Thioether was employed to bridge ferrocene and the natural anticancer product BA, which can be highly effectively triggered by the high GSH and ROS in the tumor microenvironment for the release of BA. BA induces ROS generation for chemotherapy; GOx consumes glucose to produce H_2_O_2_ and gluconic acid for starvation therapy, and all H_2_O_2_ in the TME and induced by GOx is catalyzed by ferrous ions decomposed from ferrocene to generate the highly toxic ·OH for chemodynamic therapy. Herein, thioether-bridged ferrocene-modified BA-G and the supramolecular nanoparticles GOx@BNPs were prepared, and their GSH/ROS dual-responsive drug release capability and their combination of chemodynamic/starvation/chemotherapy against cancer cells were evaluated in the present study in order to verify these hypotheses.

## 2. Results and Discussion

### 2.1. Synthesis of the Host WP6 and Guest BA-G

**Scheme 2:**
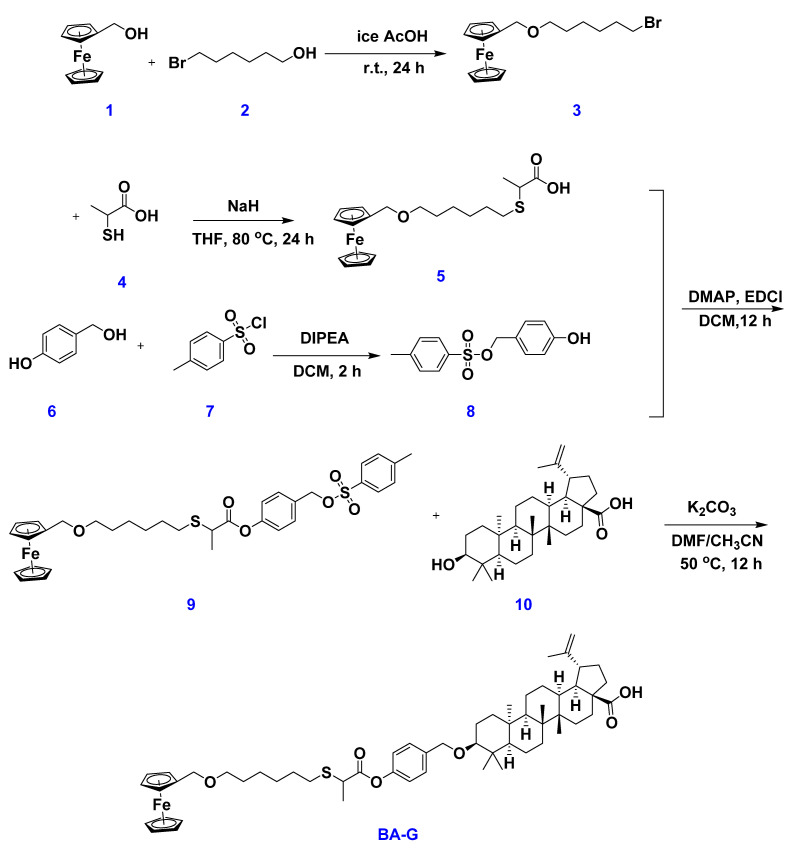
Synthetic route for the BA prodrug BA-G.

The host WP6 was synthesized as described in [42], while the BA prodrug BA-G was obtained by introducing ferrocene for binding sites, thioether as a GSH- and ROS-responsive unit, and BA as an anticancer drug. The synthetic route for guest BA-G is shown in Scheme 2.

### 2.2. Fabrication of Supramolecular Nanoparticles: BNPs and GOx@BNPs

**Figure 1:**
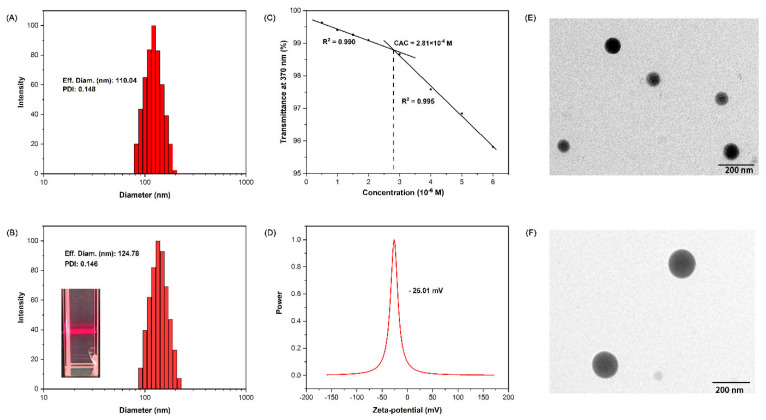
DLS data of (**A**) BNPs and (**B**) GOx@BNPs, inset: Tyndall effect of GOx@BNPs; (**C**) CAC determined by the concentration-dependent optical transmittance of BNPs at 370 nm; (**D**) *ζ*-potential of GOx@BNPs; TEM images of (**E**) BNPs and (**F**) GOx@BNPs.

BNPs were constructed through self-assembly of WP6 and BA-G. The capability to form the nanoassembly was explored based on host–guest interactions between WP6 and BA-G. The aqueous solution (containing 1% DMSO and 0.5% DMF as co-solvents, 1×10^−4^ M) of BA-G remained clear and transparent after two days, and there was no apparent Tyndall effect in the free BA-G aqueous solution. However, a legible Tyndall effect and pale blue opalescence appeared rapidly with the addition of WP6 to the above solution, confirming that WP6 can induce the self-assembly of the nanoparticle via host–guest interactions between the ferrocene moiety of BA-G and WP6. The host–guest inclusion was obtained through the ferrocene moiety of BA-G being fully threaded into the cavity of WP6 in water, in which the driving force was probably from hydrophobic interactions and π–π interactions [43]. Dynamic light scattering (DLS) measurements showed that the diameter and polydispersity (PDI) of the nanoparticles formed by WP6⊃BA-G were approximately 110.04 nm and 0.148, respectively (Figure 1A), in which the molar ratio of BA-G/WP6 was 5:1. The critical aggregation concentration (CAC) at this molar ratio was 2.81 × 10^−6^ M by the determination of the change in the transmittance at different concentrations (Figure 1C). Electrophoretic light scattering (ELS) demonstrated that the *ζ*-potential of WP6⊃BA-G nanoparticles was approximately −45.50 mV (Figure S6). In addition, these nanoparticles were very stable after 35 days (Figure S7), indicating that these nanoparticles could exist stably. Transmission electron microscopy (TEM) images indicated the formation of numerous nanoparticles, and their diameters were 98–118 nm (Figure 1E). Consequently, these WP6⊃BA-G nanoparticles with a molar ratio of BA-G/WP6 = 5:1—which have the ideal size for passive targeting and can exist stably—were chosen as candidates for further investigation, and abbreviated as BNPs.

GOx-loaded nanoparticles were constructed based on BNPs. After removing the unloaded GOx via dialysis, GOx-loaded nanoparticles were successfully obtained, denoted as GOx@BNPs. The DLS results showed that the diameter of the GOx@BNPs was 124.78 nm and their PDI was 0.146 (Figure 1B). The ζ-potential was changed to −25.01 mV (Figure 1D). TEM images indicated the morphology of GOx@BNPs to be a slight oval shape with a diameter from 113 to 136 nm (Figure 1F). Additionally, the fluorescein-isothiocyanate-labeled glucose oxidase (FITC-GOx)-loaded nanoparticles (FITC-GOx@BNPs) were prepared via the same method, and the capability of GOx loading was determined by loading FITC-labeled GOx using the same method. The maximal loading efficiency and loading content were 20.1% and 24.5%, respectively.

### 2.3. Drug Release Behavior

**Figure 2:**
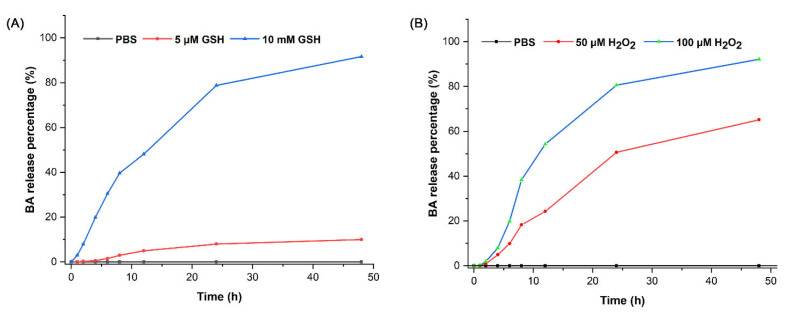
BA release profiles from the GOx@BNPs (50 µM) with various concentrations of (**A**) GSH and (**B**) H_2_O_2_ were determined via HPLC.

Because of the GSH/ROS dual-responsive thioether bond introduced in BA-G, the BA recovery and release behaviors of GOx@BNPs in mimetic normal tissue (low-expressed GSH and H_2_O_2_) or the tumor microenvironment (overexpressed GSH and H_2_O_2_) were determined via HPLC. GOx@BNPs exhibited satisfactory stability under an artificial healthy cell microenvironment (low-expressed GSH and without H_2_O_2_). As expected, the release behavior of BA was only detected in the presence of GSH or H_2_O_2_, and the release rate was positively correlated with the concentration of GSH or H_2_O_2_ (Figure 2). At the concentration of 10 mM GSH and 100 μM H_2_O_2_, the amounts of cumulative BA release were 91.68% and 92.14%, respectively, within 48 h. Meanwhile, in the artificially low-expressed GSH of the healthy cell microenvironment (5 μM), the release rate of BA was no more than 10%. This selective drug release and recovery behavior is not only conducive to avoiding damage to normal cells, but can also effectively consume the overexpressed GSH in tumor cells to improve the oxidative stress levels and enhance the CDT’s effects.

### 2.4. GOx@BNPs Induced Cascade Reactions

**Figure 3:**
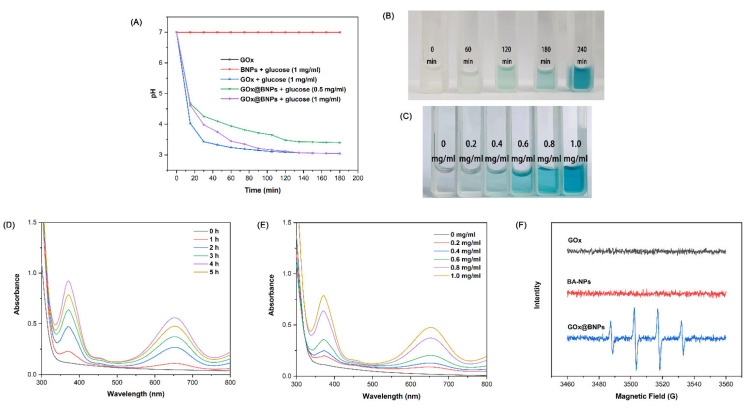
(**A**) Time-dependent pH changes of GOx@BNPs (1× 10^−4^ M) in the presence of glucose. (**B**) Photograph of GOx@BNPs (1× 10^−4^ M) in the presence of 1 mg/mL glucose from 1 to 4 h. (**C**) Photograph of GOx@BNPs (1× 10^−4^ M) in the presence of different concentrations of glucose after incubation for 4 h. (**D**) Time-dependent UV–Vis spectrum changes of GOx@BNPs (1× 10^−4^ M) in the presence of glucose (1 mg/mL) and TMB (1 mM). (**E**) UV–Vis spectrum changes of GOx@BNPs (1× 10^−4^ M) with TMB (1 mM) and different glucose concentrations after incubation for 4 h. (**F**) EPR results of different groups in the presence of glucose (1 mg/mL) for verification of ·OH production.

As described in the introduction, GOx@BNPs were intended to catalyze the conversion of glucose to gluconic acid and H_2_O_2_, and then the generated H_2_O_2_ could react with ferrocene to produce hydroxyl radicals (·OH) via cascade reaction under the acidic conditions. To validate the feasibility of cascade reactions induced by GOx@BNPs, we detected the variations in pH values and ·OH production at different levels of glucose. A rapid reduction phenomenon of pH was found in both GOx@BNPs (pH from 7.00 to 2.98) and the native GOx groups (pH from 7.00 to 3.02) in the presence of glucose (1 mg/mL). Moreover, the degree of reduction of pH values was closely related to glucose concentration (Figure 3A). By contrast, almost no apparent change in pH was monitored in the BNP group. Subsequently, the commercially available probe 3,3’,5,5’-tetramethylbenzidine (TMB), along with EPR (electron paramagnetic resonance) spectroscopy, was utilized to identify the production of ·OH. As shown, the color of the GOx@BNPs solution darkened gradually from faint yellow to blue (Figure 3B,C); in the meantime, the typical absorbance band of oxidized TMB at 652 nm enhanced drastically in the presence of glucose (without horseradish peroxidase) with increasing time or levels of glucose (Figure 3D,E). This phenomenon demonstrated efficient ·OH production by GOx@BNPs, and its yield was dependent on glucose consumption. In addition, the EPR results further proved the robust ·OH generation ability, wherein the typical 1:2:2:1 signal of ·OH was found only in the GOx@BNPs group, but not in the native GOx and BNP groups (Figure 3F). Collectively, GOx@BNPs are expected to be a promising candidate for cancer starvation and chemodynamic treatment via cascade reactions.

### 2.5. Cytotoxicity of GOx@BNPs

**Figure 4:**
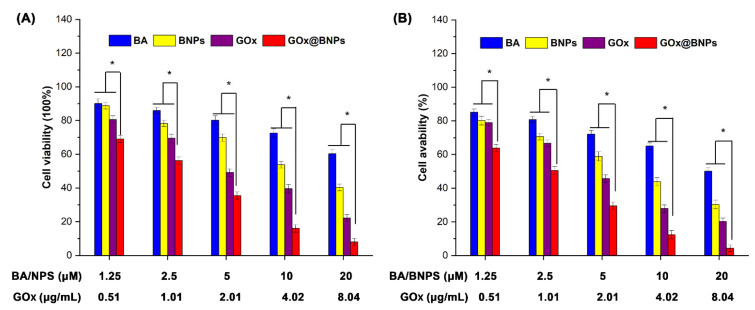
In vitro cytotoxicity of different groups against (**A**) MCF-7 and (**B**) A549 cancer cells at 37 °C for 24 h by intracellular drug delivery (* p < 0.05).

After verification the capability of GOx@BNPs to induce a cascade reaction and its cellular internalization, the cytotoxicity of GOx@BNPs against human breast cancer cells MCF-7 and human lung cancer cells A549 was evaluated by MTT assay. Considering that the glucose in the culture medium causes problems in the process of cellular internalization for GOx-containing groups, the cultured cells were washed twice with PBS after incubation for 24 h, and then incubated with glucose/serum-free media for 2 h, after which 100 μL of fresh glucose/serum-free medium containing different concentrations of BA, BNPs, GOx, or GOx@BNPs was used to treat cells for 4 h. Afterwards, any undelivered agent was fully removed by washing with PBS, before replacing the glucose-containing medium to culture for 24 h in order to determine the anticancer efficiency. As shown in Figure 4A,B, the BNP-, GOx-, and GOx@BNPs-treated groups all expressed significant proliferation inhibition ability in a concentration-dependent manner—much more potent than that of the BA-treated group. The maximum inhibition rates of GOx@BNPs against MCF-7 and A549 cancer cells were 91.99% and 95.63%, respectively, at the concentration of 20 µM—much more potent than the results of any of the other treatment groups. Since the cytotoxicity of GOx@BNPs against A549 is higher than that of MCF-7, A549 was chosen for further study.

**Figure 5:**
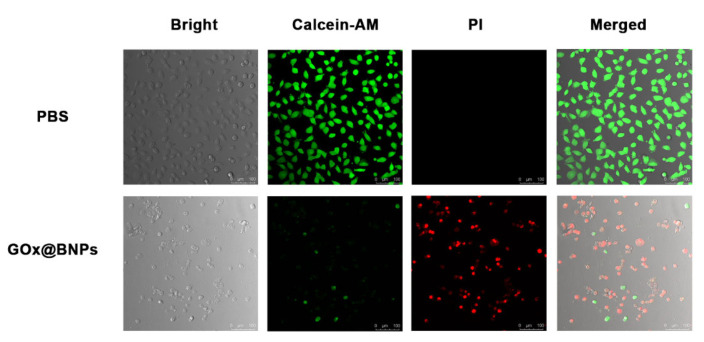
Live/dead A549 cells’ staining after treatment with PBS or GOx@BNPs (10 μM) at 37 °C for 12 h in the presence of glucose (1 mg/mL). Scale bars: 100 μm.

Furthermore, the live/dead cell staining assays were performed direct visualization of the anti-proliferative activity of GOx@BNPs against A549 cells, in which the live cells were stained as green fluorescence while the dead cells were stained as red fluorescence after treatment. As depicted in Figure 5, the PBS groups showed no detectable damage to A549 cells, but almost no live cells were found in the GOx@BNPs groups after 24 h of administration, which was consistent with the results of the MTT assays. From these results, we deduce that the outstanding anti-proliferative activity of GOx@BNPs comes from the synergistic combination of starving therapy (GOx), chemodynamic therapy (·OH), and chemotherapy (BA), as triggered by intracellular cascade reactions.

### 2.6. Cellular Internalization and Subcellullar Distribution

**Figure 6:**
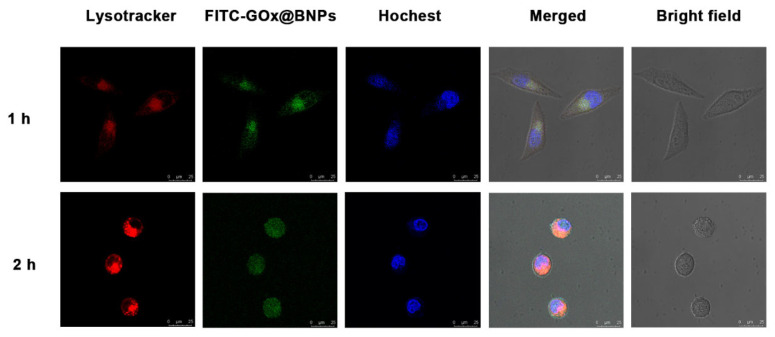
Confocal fluorescence imaging of FITC-GOx@BNPs (10 μM) in A549 cells at 37 °C for 1 h and 2 h. Scale bars: 25 μm.

FITC-GOx@BNPs in A549 cells were explored via confocal laser scanning microscopy. After co-incubation for 1 h, the intracellular green fluorescence of FITC-GOx was distinctly seen, while merged yellow fluorescence was also observed. When the co-culture was extended to 2 h, the green fluorescence was better distributed throughout the cells than after incubation for just 1 h (Figure 6). These results indicate that GOx@BNPs could be successfully internalized by cancer cells through endocytosis, enabling them to execute effective intracellular delivery for cancer treatment.

### 2.7. Mechanism of Cancer Inhibition

#### 2.7.1. Elevation of ROS

**Figure 7:**
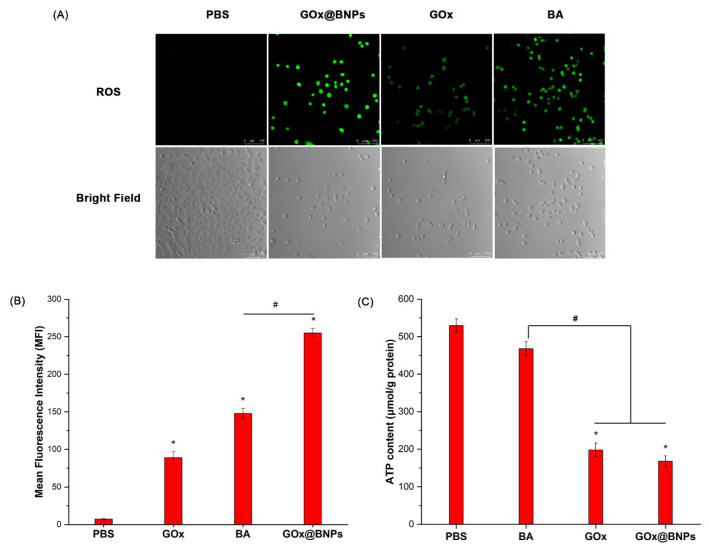
(**A**) ROS measurement in A549 cells using the DCFH-DA probe after incubation with different groups at 37 °C for 2 h. Scale bars: 100 μm. (**B**) The corresponding mean fluorescence intensity (MFI) after incubation with different groups (* *p,* ^#^ *p* < 0.05). (**C**) Intracellular ATP levels of A549 cells after treatment with different groups at 37 °C for 12 h (* *p,* ^#^ *p* < 0.05). Results are expressed as the mean ± S.D. (*n = 3*).

ROS play an important role in redox signaling pathways, which are a promising target for the treatment of multiple types of cancers [44,45,46]. Fenton-reaction-based CDT can generate ROS, which can damage the cancer cells directly [47]. To explore the inferred mechanism of cell death induced by GOx@BNPs, the levels of intracellular ROS—a significant indicator associated with CDT—were first investigated with the assistance of the ROS detector DCFH-DA. As shown in Figure 7A,B, no fluorescence signal was detected in the PBS groups; however, a very bright green fluorescence was found in the BA-, GOx-, and GOx@BNPs-treated A549 cells, and the fluorescence intensity of the GOx@BNPs-treated group was much stronger than that of both the BA- and GOx-treated groups, demonstrating the much more efficient ·OH production ability of GOx@BNPs in A549 cancer cells.

#### 2.7.2. Decrease in ATP

As mentioned above, GOx could selectively catalyze the oxidation of glucose into gluconic acid and H_2_O_2_ with high efficiency; hence, the depletion of intratumoral glucose by GOx can effectively consume the energy for tumor growth and starve tumors to death. We further measured the intracellular ATP content, which can effectively reflect the effects of starvation therapy. As shown in Figure 7C, the intracellular ATP concentration was 167.44 μmol/g protein after administration of GOx@BNPs, which is lower than that of GOx (197.78.23 μmol/g protein), and much lower than those of PBS (529.60 μmol/g protein) and BA (468.06 μmol/g protein) groups. This remarkable ATP consumption behavior confirms the starvation therapy effect of GOx@BNPs on cancer cells.

#### 2.7.3. Evaluation of Mitochondrial Transmembrane Potential (MTP)

**Figure 8:**
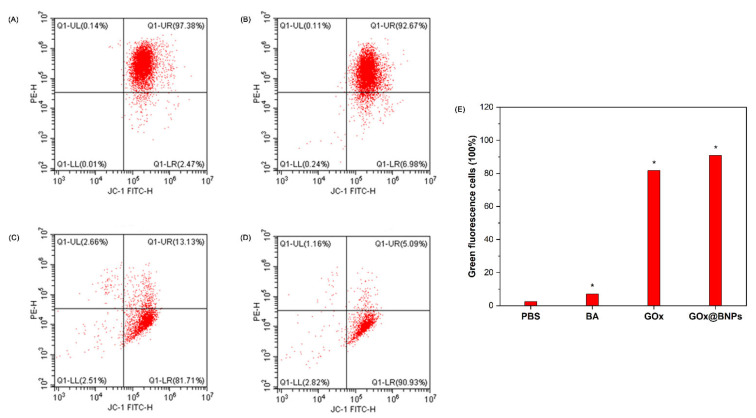
Representative flow cytometric analysis of JC-1-stained A549 cells treated with (**A**) PBS; (**B**) BA; (**C**) GOx; and (**D**) GOx@BNPs. (**E**) Statistical analysis of A549 cells treated with different groups (* *p* < 0.05). A549 cells were treated with different groups for 24 h, and then incubated with JC-1 at 37 °C for 15 min and, finally, detected via flow cytometry. The decrease in MTP is indicated by an increase in the percentage of green fluorescence cells.

The MTP of A549 in the GOx@BNPs-treated group was assessed after staining with JC-1 using flow cytometric analysis. In normal mitochondria, JC-1 aggregates in the mitochondrial matrix to form J-aggregate, which exhibits a strong red fluorescence; however, it will emit green fluorescence at a low MTP [48]. After incubation with different groups for 24 h, A549 cells were then incubated with JC-1 at 37 °C for 15 min and, finally, detected via flow cytometry. The mitochondria of healthy cells containing red JC-1 aggregates were detected by the FL2 channel, while the mitochondria of damaged cells containing the green JC-1 monomers were detected by the FL1 (FITC) channel. The decrease in MTP is indicated by an increase in the percentage of green fluorescence cells. As shown in Figure 8, the percentages of green fluorescence cells in the BA-, GOx-, and GOx@BNPs-treated groups were much higher than that of PBS-treated group, demonstrating that the MTP was significantly decreased. Among them, the MTP of the GOx@BNPs-treated group was the lowest, with a much more potent decrease than that of the BA- and GOx-treated groups.

#### 2.7.4. Induction of Apoptosis

**Figure 9:**
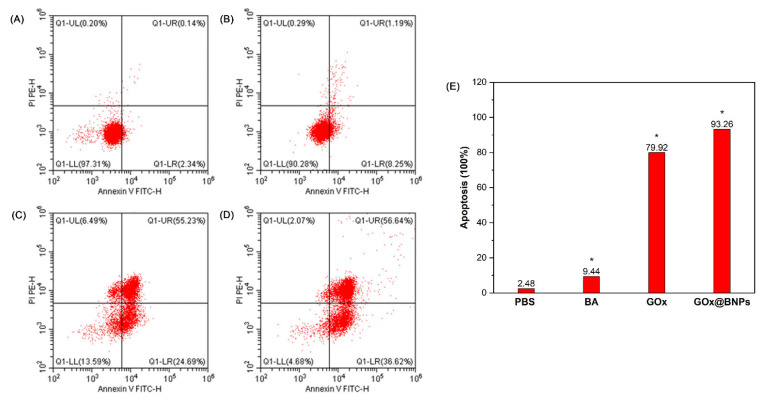
Representative graphs and statistical analysis of Annexin V-FITC/PI staining. Flow cytometric analysis of Annexin V-FITC/PI stained A549 cells treated with (**A**) PBS; (**B**) BA; (**C**) GOx; and (**D**) GOx@BNPs. (**E**) Statistical analysis the apoptosis rates of A549 cells at 37 °C for 12 h. Inserted numbers in the profiles indicate the percentage of the cells present in this area. Date are expressed as the mean ± S.D., *n* = 3 (* p < 0.05).

BA can act as an apoptosis activator to exert an anticancer effect [49]. Eventually, the apoptosis of A549 cancer cells treated with different groups was evaluated via flow cytometry. As shown in Figure 9, the BA-, GOx-, and GOx@BNPs-treated groups all significantly induced cell apoptosis compared to the PBS-treated group. The apoptosis rates of both the GOx@BNPs- (93.26%) and GOx (79.92%)-treated groups were much higher than that of the BA-treated group (9.44%). Among the various groups, GOx@BNPs induced the highest apoptosis rate through intracellular drug delivery.

As mentioned above, this work presents a novel method to regulate the tumor microenvironment for improved CDT and the preparation of novel GSH/ROS dual-responsive supramolecular nanoparticles, which can exert significant cytotoxicity against cancer cells through the synergistic interaction of chemodynamic therapy, starvation therapy, and chemotherapy. For example, BA can induce elevated ROS in cancer cells, as is clearly demonstrated in Figure 7A,B. Compared to the PBS-treated group, a very bright green fluorescence was found in the BA- and GOx-treated groups, and the fluorescence intensity of the GOx@BNPs-treated group was much stronger than that of both the BA- and GOx-treated groups, demonstrating that GOx@BNPs induced potent ROS elevation in A549 cancer cells through the synergistic interaction of the chemotherapy of BA and the Fenton-reaction-based chemodynamic therapy. The preliminary research mechanism shows that the dramatic suppression of cancer cell growth induced by GOx@BNPs was derived from the elevation of ROS, decrease in ATP and MTP and, finally, cell apoptosis. However, in order to explain the findings better, more related mechanisms must be explored in the remainder of this study. Although this kind of nanoparticle has the ideal size for passive targeting, it is still better to introduce an active targeting group in the supramolecular nanoparticles so as to enable active targeting of the nanoparticles for further study. In an effort to verify whether the GOx@BNPs could specifically respond to the overexpressed GSH and ROS in the TME, the cytotoxicity of GOx@BNPs against human normal cells, as well as their antitumor activity in vivo, will be evaluated in the remainder of this study.

## 3. Materials and Methods

### 3.1. General Information

All reagents were purchased commercially and used without further purification unless otherwise noted. All reactions were performed in a normal air atmosphere unless otherwise stated. Glucose oxidase (GOx, 100 unit/mg protein) was purchased from Sigma-Aldrich. Column chromatography was performed with silica gel (200–300 mesh) produced by Qingdao Marine Chemical Factory, Qingdao (China). NMR spectra were recorded on a Bruker DPX 400 MHz spectrometer using the internal standard tetramethylsilane (TMS). HRMS data were recorded on a JMS-SX102A (FAB) or via LC/MSD TOF. Dynamic light scattering (DLS) measurements were taken using a NanoBrook 90Plus Zeta (Brookhaven Instruments Corporation, New York, NY, USA) with a 200 mW polarized laser source (λ = 514 nm). Zeta potential measurements were recorded at 25 °C using a NanoBrook 90Plus Zeta (Brookhaven Instruments Corporation, New York, NY, USA). Transmission electron microscope (TEM) imaging was performed using a HITACHI HT-7700 instrument. The UV–Vis absorption spectra were recorded on a spectrometer (UV1800PC, Jinghua, Shanghai, China). The EPR (electron paramagnetic resonance) spectra were recorded on JON Bruker BioSpin GmbH. The excitation and emission spectra were measured on a SHIMADZU RF-5301PC fluorescence spectrometer.

### 3.2. Preparation of BA-G

#### 3.2.1. Synthesis of Compound 3

Ferrocene methanol **1** (1.0803 g, 5 mmol), 2,2’-(ethane-1,2-diylbis(oxy))bis(ethan-1-ol) **2** (3.0034 g, 20 mmol), and Al(OTf)_3_ (48 mg, 0.1 mmol) were suspended in anhydrous dichloromethane. After the mixture was stirred at ambient temperature for 6 h, an appropriate amount of 5% Na_2_CO_3_ was then added, and the mixture was extracted with CH_2_Cl_2_ (3 times with 10 mL). The combined organic layer was washed with brine, dried with anhydrous sodium sulfate, and filtered, and the solvent was removed via evaporation in vacuum. The crude products were purified via column chromatography using ethyl acetate/petroleum ether (1:5, *v/v*) as an eluent to produce target compound **3** as a clear, pale yellow oil (1.2188 g, 3.5 mmol, 70%). ^1^H NMR (400 MHz, CDCl_3_), δ 4.34 (d, *J* = 4.7 Hz, 2H), 4.24–4.23 (m, 2H), 4.17–4.11 (m, 7H), 3.74–3.70 (m, 2H), 3.68–3.57 (m, 10H). The ^1^H NMR spectra of compound **3** are available in Figure S1.

#### 3.2.2. Synthesis of Compound 5

NaH (0.6 g, 25 mmol) and compound **4** (0.53 g, 5 mmol) were suspended in anhydrous THF under a nitrogen atmosphere and stirred for 30 min at 80 °C. Then, compound **3** (2 g, 5.5 mmol) dissolved in anhydrous THF was added to the suspension, and the reaction mixture was continuously stirred for 24 h at 80 °C. After the mixture was cooled to room temperature, a large amount of water was added to quench the reaction. The water layer was first extracted with EA and then acidified to pH 5–6 with 2 mol/L hydrochloric acid, and finally extracted with EA (3 times with 30 mL). The combined organic layer was washed with brine, dried with anhydrous sodium sulfate, and filtered, and the solvent was removed via evaporation in vacuum to produce target compound **5** as a clear, pale yellow oil (1.4252 g, 3.5 mmol, 70%).

#### 3.2.3. Synthesis of Compound 8

Compound **6** (0.62 g, 5 mmol) and DIPEA (1.65 mL, 10 mmol) were dissolved in anhydrous dichloromethane, and then compound **7** (1.14 g, 6 mmol) was added in an ice bath. The mixture was stirred for 2 h at 25 °C and extracted with CH_2_Cl_2_ (3 times with 10 mL). The combined organic layer was washed with brine, dried with anhydrous sodium sulfate, and filtered, and the solvent was removed via evaporation in vacuum. The crude products were purified via column chromatography using ethyl acetate/petroleum ether (1:5, *v/v*) as an eluent to produce target compound **8** as a clear, pale yellow oil (1.11 g, 4.0 mmol, 80%).

#### 3.2.4. Synthesis of Compound 9

Compound **5** (70 mg, 0.17 mmol), EDCI (65.2 mg, 0.34 mmol), and DMAP (4.15 mg, 0.034 mmol) were suspended in anhydrous dichloromethane. The mixture was stirred at room temperature for 30 minutes, then compound **8** (57 mg, 0.2 mmol) dissolved in anhydrous dichloromethane was slowly added. The reaction mixture was further stirred at room temperature for 12 h, and then extracted with dichloromethane. The organic layer was washed with brine, dried with anhydrous sodium sulfate, and filtered, and the solvent was removed via evaporation in vacuum. The crude products were purified by column chromatography using ethyl acetate/ petroleum ether (1:9, *v/v*) as an eluent to produce target compound **9** as a clear, pale yellow oil (80 mg, 0.12 mmol, 70%). ^1^H NMR (400 MHz, CDCl_3_), δ 7.64 (d, *J* = 8.3 Hz, 2H), 7.26–7.20 (m, 4H), 6.91 (d, *J* = 8.6 Hz, 2H), 5.04 (d, *J* = 4.9 Hz, 2H), 4.19 (s, 2H), 4.15 (s, 2H), 4.05 (s, 7H), 3.34–3.30 (m, 3H), 2.46 (d, *J* = 7.3 Hz, 2H), 2.38 (s, 3H), 1.45 (s, 4H), 1.37 (d, *J* = 7.1 Hz, 3H), 1.23–1.19 (m, 6H). The ^1^H NMR spectra of compound **9** are available in Figure S2.

#### 3.2.5. Synthesis of Copound BA-G

Betulinic acid (0.47 g, 1 mmol), compound **9** (0.80 g, 1.2 mmol), and K_2_CO_3_ (0.28 g, 2 mmol) were suspended in anhydrous DMF and anhydrous acetonitrile, and then the mixture was stirred for 12 h at 50 °C. After the reaction mixture was allowed to cool to room temperature, an appropriate amount of cold water was added, and the white solid was precipitated and filtered, washed with PE, and dried in vacuum, and the product was formed as a pale yellow solid (0.81 g, 0.85 mmol, 85%).^1^H NMR (400 MHz, CDCl_3_), δ 4.66 (s, 1H), 4.53 (s, 1H), 4.19 (s, 2H), 4.16 (s, 2H), 4.06 (s, 7H), 3.99 (d, *J* = 9.0 Hz, 2H), 3.35–3.32 (m, 2H), 3.11 (d, *J* = 6.2 Hz, 1H), 2.93 (d, *J* = 4.6 Hz, 1H), 1.80 (d, *J* = 9.8 Hz, 4H), 1.52 (d, *J* = 11.5 Hz, 12H), 1.29 (s, 16H), 1.18 (s, 6H), 0.89 (s, 7H), 0.84–0.77 (m, 9H), 0.75 (s, 3H), 0.69 (s, 3H). ^13^C NMR (101 MHz, CDCl_3_), δ 175.2, 149.6, 108.5, 77.9, 68.8, 68.4, 68.1, 67.4, 62.9, 55.5, 54.3, 49.5, 48.4, 46.0, 41.4, 39.7, 37.8, 37.7, 37.3, 36.2, 36.0, 33.3, 31.2, 29.6, 28.6, 27.7, 26.9, 26.4, 24.9, 24.8, 24.5, 19.9, 18.3, 17.3, 15.1, 14.9, 14.3, 13.7. HRMS *m/z* 949.5103 [M + H] ^+^(calculated for C_15_H_15_O^+^, 949.5103). The NMR and HRMS spectra of BA-G are available in Figures S3–S5.

### 3.3. Preparation of BNPs and GOx@BNPs

BNPs were constructed through self-assembly of WP6 and BA-G. Typically, BA-G was dissolved in 300 μL of DMSO/MDF solution (*v/v* = 2:1) to obtain a stock solution (1 × 10^−2^ M). Then, 45 μL of BA-G stock solution was rapidly injected into WP6 aqueous solution with a 5:1 ratio of BA-G and WP6 to obtain 3 mL solutions. The mixtures were shaken slowly for 5 minutes and then left to stand overnight to form stable supramolecular BNP nanoparticles.

GOx-loaded nanoparticles were constructed based on BNPs. Briefly, 300 μL of 2000 μg/mL GOx solution were added to the aqueous solution containing WP6. Subsequently, 45 μL of DMSO/DMF stock solution (*v/v* = 2:1) was added to BA-G to obtain 3 mL ultimate solutions (the ultimate concentration of BA-G was 1 × 10^−4^ M, and the ultimate concentration of GOx was 200 μg/mL). The mixtures were shaken for 5 minutes and then left to stand overnight. To remove organic solvents and unloaded GOx, the mixtures were further purified via dialysis (molecular weight cutoff 300000). The GOx-loaded nanoparticles were successfully prepared and denoted as GOx@BNPs.

Furthermore, we also prepared FITC-GOx-loaded nanoparticles according to similar procedures, and used fluorescence spectroscopy to quantify the GOx loading capability.

The GOx loading content (wt %) and loading efficiency (%) were calculated by the following equation:

Loading content (wt %) = (*m_GOx_*_-loaded_/*m_GOx_*_-loaded_ + *m_WP6_ + m_BA-G_*) × 100

Encapsulation efficiency (%) = (*m_GOx_*_-loaded_/*m_GOx_*) × 100

where *m*_*GOx*-loaded_, *m_GOx_*, *m_WP6_*, and *m_BA-G_* are the mass of GOx encapsulated into the vesicles and the mass of GOx, WP6, and BA-G added, respectively. The mass of GOx was measured via fluorescence spectroscopy at 520 nm and calculated according to a standard calibration curve of GOx in deionized water, with concentrations from 10 to 100 μg/mL.

### 3.4. Synthesis of FITC-GOx

*FITC-GOx* was prepared as described in [50]. Briefly, FITC solution (10 mg/mL in anhydrous DMSO, 25 μL) was added to 1 mL aqueous GOx solutions (10 mg/mL containing 100 mM sodium carbonate). The reaction was further incubated at 4 °C overnight, followed by dialysis with deionized water overnight in the dark and, finally, lyophilization.

### 3.5. In Vitro Release of BA

Briefly, BNP solution was treated with varying concentrations of GSH (0 or 5 μM, or 10 mM) or H_2_O_2_ (0, 50, or 100 μM) at 37 °C. Twenty microliters of the mixture solution (0.5 × 10 ^−4^ M) was further taken for HPLC measurement at different timepoints (0, 1, 2, 4, 6, 8, 12, 24, and 48 h). The released BA was detected via HPLC (SHIMADZU, Kyoto, Japan) using 90% methanol and 10% water containing 0.1% phosphoric acid as a mobile phase, with a flow rate of 1 mL min^−1^ (Agilent XDB C18 reversed-phase (150 mm × 4.6 mm, 5 μm); temperature, 25 °C; UV detector at 210 nm).

### 3.6. pH Variation under Different Concentrations of Glucose

Different amounts of glucose (0, 0.5, or 1 mg/mL) were added to GOx@BNPs (1 × 10^−4^ M, in which the GOx concentration was 40.2 μg/mL) aqueous solution (pH = 7.0), and the pH values were monitored using a pH meter at different timepoints. Furthermore, the GOx (40.2 μg/mL) and BNPs (1 × 10^−4^ M) with no glucose were set as control groups.

### 3.7. Measurement of ·OH Generation

3,3’,5,5’-Tetramethylbenzidine (TMB) was used to investigate the generation of ·OH through UV–Vis spectra. With TMB oxidized by ·OH, the change in absorbance intensity at 665 nm in solutions treated with different formulations was monitored at 37 °C. In addition, the ·OH generation was also monitored via electron paramagnetic resonance (EPR) (Bruker, Karlsruhe, German) with the ·OH-trapping probe 5, 5-dimethyl-1-pyrroline N-oxide (DMPO) (Sigma-Aldrich, St. Louis, MO, USA).

Firstly, the time-dependent ·OH generation was investigated: 25 μL of TMB (10.6 mg/mL) in DMSO and 50 μL of aqueous glucose solution (24 mg/mL) were added to a 1 mL GOx@BNPs solution to obtain a mixed suspension (BA-G 100 μM, TMB 1 mM, GOx 40.2 μg/mL, glucose 1 mg/mL), and the ·OH-induced TMB oxidation was monitored at different timepoints.

Secondly, the glucose-dependent ·OH generation was further investigated: 25 μL of TMB (10.6 mg/mL) in DMSO and aqueous glucose solutions of various concentrations (0, 10, 20, 30, 40, or 50 μL; 20 mg/mL) were added to 1 mL GOx@BNPs solutions to obtain a mixed suspension (with the same BA-G concentration of 100 μM, the same TMB concentration of 1 mM, and the same GOx concentration of 40.2 μg/mL), and the ·OH-induced TMB oxidation was tested by recording the change in the absorbance value at 665 nm via UV–Vis spectroscopy at 37 °C for 4 h.

Finally, the EPR results further proved the robust ability of ·OH generation. Briefly, 100 µL of the prepared glucose solution (10 mg/mL) was added to 1 mL of GOx (40.2 μg/mL), BNPs (100 µM), or GOx@BNPs (with the same BA-G concentration of 100 μM and the same GOx concentration of 40.2 μg/mL). After incubation at 37 °C for 4 hours, DMPO was added, and the ·OH generation was monitored via EPR.

### 3.8. In Vitro Cytotoxicity by Intracellular Drug Delivery

The *in vitro* cytotoxicity of GOx@BNPs against MCF-7 human breast cancer cells and A549 human lung cancer cells were evaluated by MTT assay. Briefly, the cancer cells were seeded in 96-well plates at a density of 1 × 10^4^ cells per well in 100 μL of DMEM containing 10% fetal bovine serum (Gibco), and cultured in 5% CO_2_ at 37 °C for 24 h. Then, cultured cells were washed twice with PBS, and incubated with glucose/serum-free media for 2 h. Next, 100 μL of fresh glucose/serum-free media containing different concentrations of BA, BNPs, GOx, or GOx@BNPs were used to treat cells for 4 h. Undelivered sample was gently washed out at once with PBS, and the cells were further incubated with glucose/serum-free media for 30 min. Then, the cells were incubated with glucose-containing media for 24 h. Subsequently, 10 μL of MTT solution (5 mg/mL) was added into each well and incubated for another 4 h. After that, the media containing MTT were removed, and dimethyl sulfoxide (DMSO, 100 μL) was added to each well to dissolve the MTT formazan crystals. Finally, the plates were shaken for 30 min, and the absorbance of the formazan product was measured at 490 nm using a microplate reader (Thermo Scientific, Waltham, MA, USA). Untreated cells in media were used as blank controls. All experiments were carried out with five replicates. The cytotoxicity was expressed as the percentage of cell viability relative to the blank control.

### 3.9. Live/Dead Cell Staining

The live/dead cell staining experiment was carried out via CLSM. After being cultured for 24 h in 96-well plates, A549 cells were incubated with PBS and GOx@BNPs (with the same BA concentration of 10 μM) for 12 h via intracellular drug delivery. The A549 viability was tested after cell death with 2 μM calcein AM and 4 μM PI for 30 min via CLSM (green channel: λex = 488 nm, λem = 500–530 nm; red channel: λex = 535 nm, λem = 600–630 nm).

### 3.10. Cellular Uptake and Subcellular Distribution

The cellular uptake and subcellular distribution of FITC-GOx-loaded vesicles (FITC-GOx@NPs) were examined in A549 cancer cells. Briefly, A549 cells were seeded in a confocal dish at a density of 1 × 10^5^ cells and cultured in 1.0 mL of complete RPMI-1640 culture medium for 24 h before treatment. Then, the original medium was removed and cells were incubated with glucose/serum-free medium for 2 h. Next, cells were treated with FITC-GOx@NPs (10 μM) for 1 h or 2 h. Then, the culture medium was removed and cells were washed thrice with PBS. Thereafter, the LysoTracker Red DND-99 (Thermo Fisher, Waltham, MA, USA) was added to the medium at a final concentration of 75 nM for 1 h to label lysosomes. Next, the cells were washed with PBS three times, and Hoechst 33342 (Thermo Fisher, Waltham, MA, USA) was added to the medium at a final concentration of 1 μg/mL for 15 min to stain nuclei. Finally, the culture medium was removed, and the cells were washed thrice with PBS, after which the cells were fixed in 4% paraformaldehyde for 20 min and washed three times with PBS. Fluorescence microscopy (Leica TCS SP8, Weztlar, German) was used to investigate the cellular uptake and intracellular localization. The fluorescence characteristics of FITC-GOx-loaded vesicles were used to directly monitor the localization of GOx without utilizing additional dye.

### 3.11. Intracellular Reactive Oxygen Species (ROS) Detection

The intracellular generation of ROS was determined using the ROS probe 2′,7′-dichlorodihydrofluorescein diacetate (DCFH-DA). The A549 cells were seeded at a density of 5 × 10^5^ cells in a confocal dish and incubated with 1 mL of complete RPMI-1640 medium at 37 °C in 5 % CO_2_ for 24 h. The medium was replaced with fresh RPMI-1640 containing PBS, GOx (4.02 μg/mL), BA (10 µM), or GOx@BNPs (10 µM, in which the GOx concentration was 4.02 μg/mL). After 2 h, DCFH-DA (10 µM) was added and incubated for 30 min. Then, the cells were washed three times with PBS and imaged via CLSM. DCF decomposed from DCFH-DA was excited at 488 nm, and fluorescence was detected from 510 to 550 nm.

### 3.12. Intracellular ATP Level Measurements

The ATP levels in A549 cells were measured using an ATP assay kit (Nanjing Jiancheng Bioengineering Institute, Nanjing, China). Briefly, A549 cells were seeded in a six-well plate at a density of 80,000 cells per well and cultured for 24 h. The cells were incubated with PBS, GOx (4.02 μg/mL), BA (10 µM), or GOx@BNPs (10 µM, in which the GOx concentration was 4.02 μg/mL) for 6 h. Next, the recommended procedures of the kit were used to determine the ATP levels in cells.

### 3.13. Evaluation of Mitochondrial Transmembrane Potential (MTP)

A549 cells were seeded in 6-well cell culture plates (Corning Incorporated, New York, NY, USA) at a density of 5 × 10^4^ cells per well in 1 mL of 90% F12K and 10% FBS medium, and cultured at 37 °C in 5% CO_2_ for 24 h. Then, the original medium was removed, and the cells were incubated with glucose-free 1640 medium. PBS, GOx (4.02 µg/mL), BA (10 µM), and GOx@BNPs (10 µM, in which the GOx concentration was 4.02 μg/mL) were dissolved in glucose/serum-free media at 37 °C for 24 h. A549 cells incubated with PBS were used as controls. After incubation for 24 h, the cell solutions were trypsinized and centrifuged at 2000 rpm for 5 min. The culture medium was removed, and the cells were washed twice with cold PBS. After removal of the supernatants, the cells were resuspended in incubation buffer (500 μL) and then incubated with a mitochondrial transmembrane potential (MTP) detection kit (Jiangsu KeyGEN Biotech Corp., Ltd, Nanjing, China) according to the manufacturer’s protocol: JC-1 was added to the cell suspensions, and after incubation in the dark for 15 min, the percentage of green fluorescence cells was detected via flow cytometry using a Becton Dickinson FACSCalibur flow cytometer. The decrease in MTP was indicated by an increase in the percentage of green fluorescence cells.

### 3.14. Flow Cytometric Analysis of Apoptosis

A549 cells were seeded in 6-well cell culture plates (Corning Incorporated, New York, NY, USA) at a density of 5 × 10^4^ cells per well in 1 mL of complete RPMI 1640 medium, and cultured at 37 °C in 5% CO_2_ for 24 h. Then, the original medium was removed, and cells were incubated with glucose-free 1640 medium. PBS, GOx (4.02 µg/mL), BA (10 µM), and GOx@BNPs (10 µM, in which the GOx concentration was 4.02 μg/mL) were dissolved in glucose/serum-free media at 37 °C for 24 h. A549 cells incubated with PBS were used as controls. After incubation for 24 h, the cell solutions were trypsinized and centrifuged at 2000 rpm for 5 min. The culture medium was removed and the cells were washed twice with cold PBS. After removal of the supernatants, the cells were resuspended in binding buffer (500 μL). The apoptotic cells were determined by staining using an Annexin V-FITC/PI apoptosis detection kit (Jiangsu KeyGEN Biotech Corp., Ltd, Naijing, China) according to the manufacturer’s protocol: Annexin V-FITC (5 μL) and propidium iodide (5 μL) were added to the cell suspensions, and after incubation in the dark for 15 min, cell apoptosis was detected via flow cytometry using a Becton Dickinson FACSCalibur flow cytometer, and 5 × 10^5^ cells were tested for each sample.

### 3.15. Statistical Analysis

The statistical analysis of different groups was compared using Student’s *t*-test. *p*-values on graphs are denoted within each figure panel as * *p* < 0.05 or ^#^ *p* < 0.05 (significant).

## 4. Conclusions

In summary, GSH/ROS dual-responsive novel supramolecular nanoparticles (GOx@BNPs) for chemo–chemodynamic combination therapy were developed based on water-soluble pillar[6]arene and a thioether-bridged ferrocene-modified natural anticancer BA prodrug, followed by encapsulation of GOx in the nanoparticles. The encapsulation of GOx in the nanoparticles could first catalyze the conversion of glucose into gluconic acid and H_2_O_2_, and then the generation of H_2_O_2_ further reacted with ferrocene to produce highly active ·OH in succession via the Fenton reaction. The novel supramolecular nanoparticles could be activated by the overexpressed GSH and ROS in the TME, which not only improved the BA recovery and release capability of GOx@BNPs, but also enhanced the Fenton reaction to produce ·OH and exert potent cytotoxicity in cancer cells. Investigation of antitumor activity and mechanisms revealed that the dramatic suppression of A549 cancer cell growth induced by GOx@BNPs was derived from the elevation of ROS levels, the decrease in ATP and MTP and, finally, the induction of cell apoptosis through the synergistic interaction of CDT, starvation therapy, and chemotherapy via an intratumoral cascade reaction. This work presents a novel method to regulate the tumor microenvironment for improved CDT and the preparation of novel GSH/ROS dual-responsive supramolecular nanoparticles based on pillar[6]arene and a BA prodrug for the combination of CDT, starvation therapy, and chemotherapy, which may become a promising strategy for cancer treatment, and warrants intensive study.

## Data Availability

Not applicable.

## Supplementary Materials

The following are available online: Figure S1: ^1^HNMR spectra of compound **3**; Figure S2: ^1^HNMR spectra of compound **9**; Figure S3: ^1^HNMR spectra of BA-G; Figure S4: ^13^CNMR spectra of BA-G; Figure S5: HRMS of BA-G; Figure S6: Zeta potential of BNPs; Figure S7: Stability of the BNPs and GOx@BNPs.
