# Supplementary material for: GSH/ROS Dual-Responsive Supramolecular Nanoparticles Based on Pillar[6]arene and Betulinic Acid Prodrug for Chemo–Chemodynamic Combination Therapy
Source: Molecules. 2021 Sep 29;26(19):5900. doi: 10.3390/molecules26195900 (PMC8512399; doi:10.3390/molecules26195900)
Supplement: Supplementary file 1 [file molecules-26-05900-s001.zip › molecules-1401889-supplementary.pdf]

GSH/ROS Dual-Responsive Supramolecular Nanoparticles based on Pillar[6]arene and Betulinic Acid Prodrug for Chemo-Chemodynamic Combination Therapy

Peng Zhu [1,2], Weidan Luo [1], Jianqiang Qian [2], Chi Meng [2], Wenpei Shan [2], Zhongyuan Xu [2], Wei Zhang [1], Xin Liu [2,*] and Yong Ling [2,*]

[1] State Key Laboratory of Quality Research in Chinese Medicine, Macau Institute for Applied Research in Medicine and Health, Macau University of Science and Technology, Taipa, Macau, China; zp1216834231@gmail.com (P.Z.); luoweidan111@163.com (W.L.); wzhang@must.edu.mo (W.Z.)

[2] School of Pharmacy and Jiangsu Province Key Laboratory for Inflammation and Molecular Drug Target, Nantong University, Nantong 226001, China; qiq1273752353@163.com (J.Q.); jsxzmc123456@163.com (C.M.); swp1902@163.com (W.S); xzy424052148@163.com (Z.X.)

* Correspondence: Lyyy111@sina.com (Y.L.); xinliunju@foxmail.com (X.L.)

Supporting Information:

Figures

Figure S1: ^{1}HNMR spectra of compound **3** acquired at 400 MHz in $CDCl_3$.

Figure S2: ^{1}HNMR spectra of compound **9** acquired at 400 MHz in $CDCl_3$.

Figure S3: ^{1}HNMR spectra of compound BA-G acquired at 400 MHz in $CDCl_3$.

Figure S4: ^{13}CNMR spectra of compound BA-G acquired at 101 MHz in $CDCl_3$.

Figures S5: HRMS of BA-G.

Figures S6: Zeta-potential of BNPs nanoparticles.

Figures S7: Stability of the BNPs and GOx@BNPs.

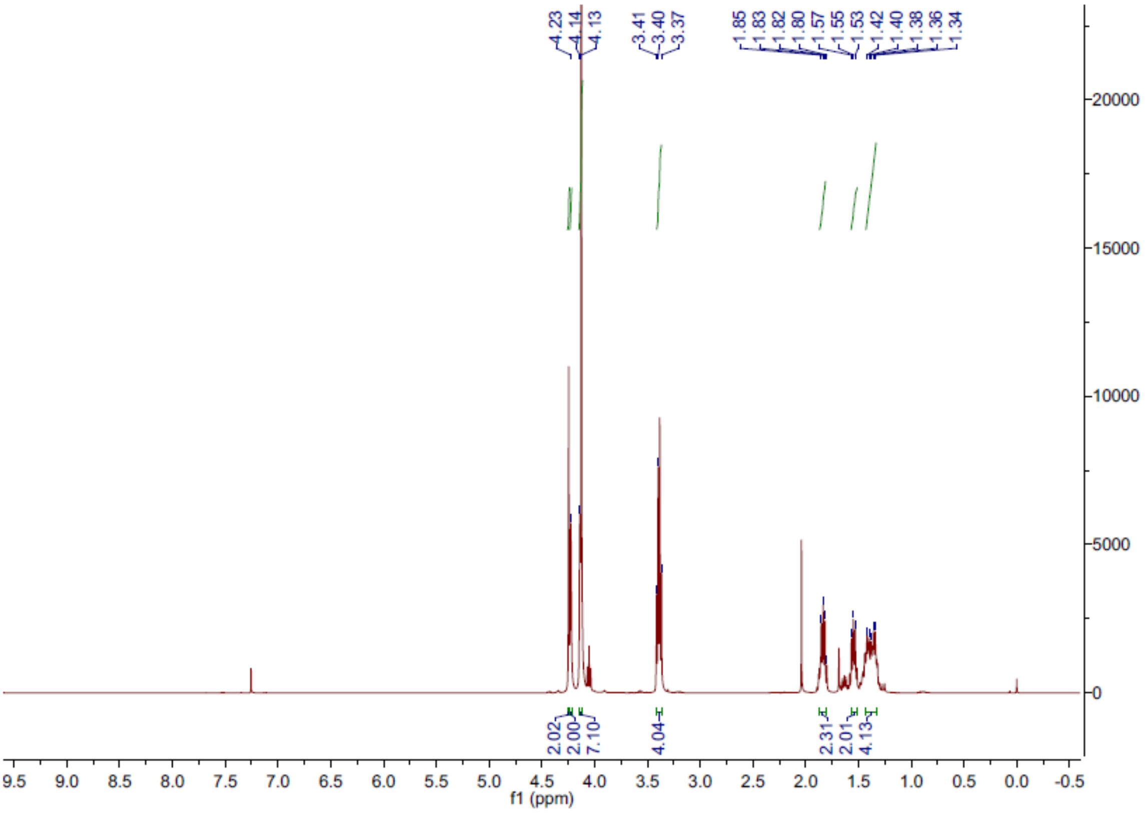

Figure S1. ^{1}H NMR spectrum (400 MHz, $CDCl_3$, 298 K) of compound **3**.

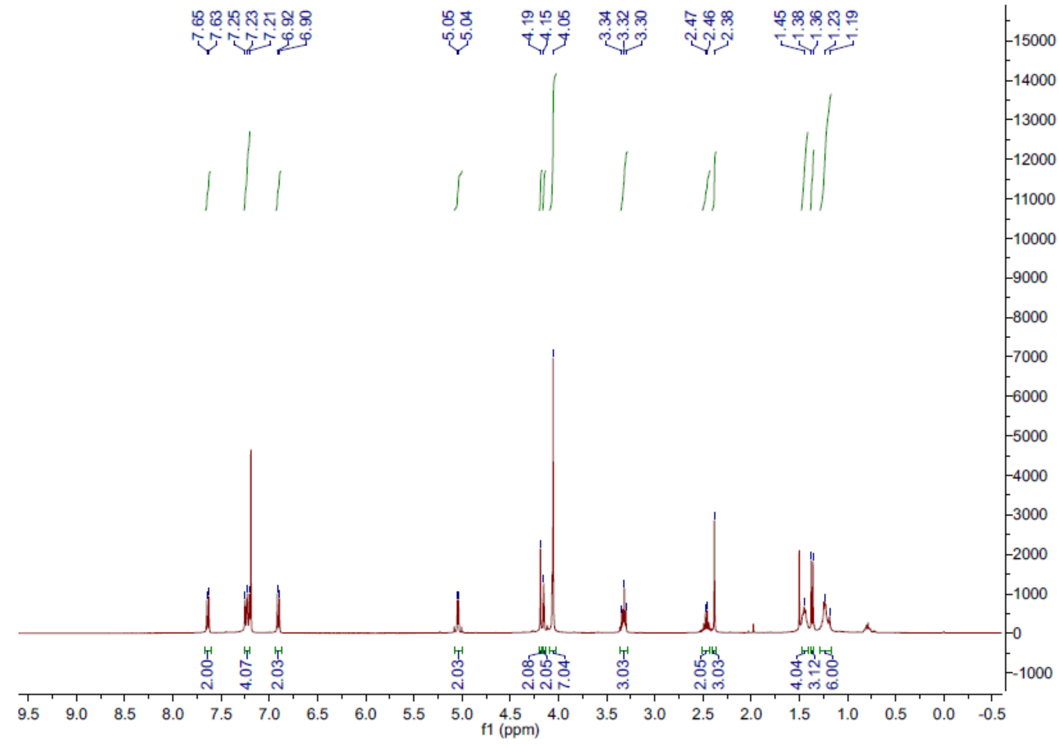

Figure S2. ^{1}H NMR spectrum (400 MHz, $CDCl_3$, 298 K) of compound **9**.

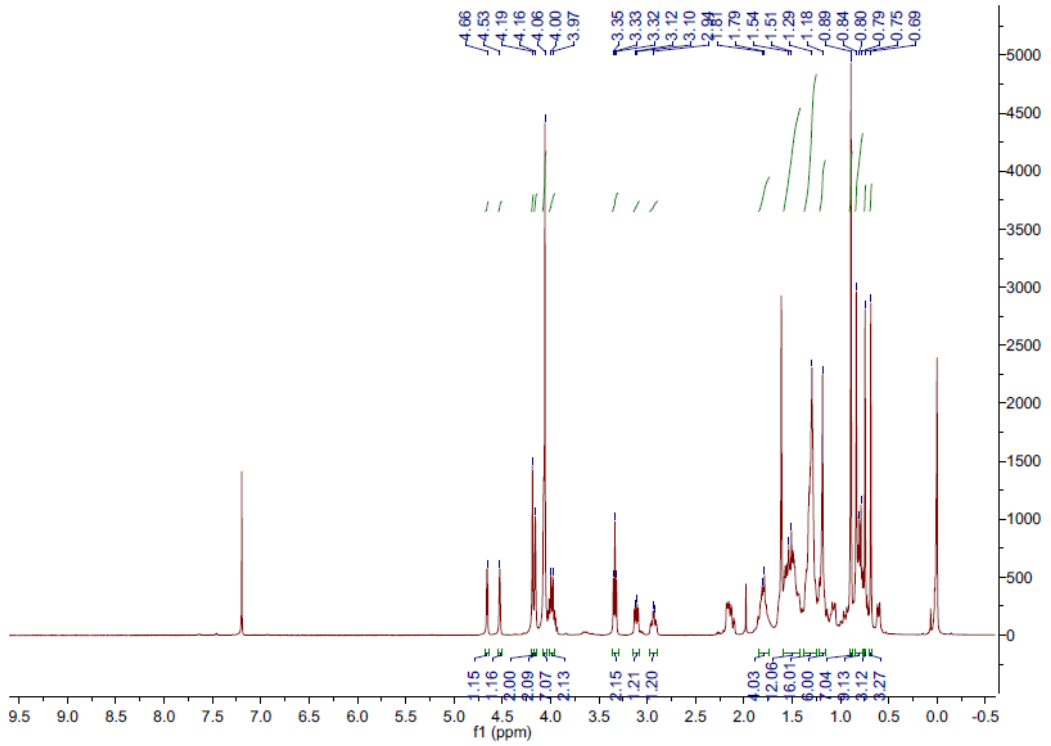

Figure S3. ^{1}H NMR spectrum (400 MHz, $CDCl_3$, 298 K) of compound BA-G.

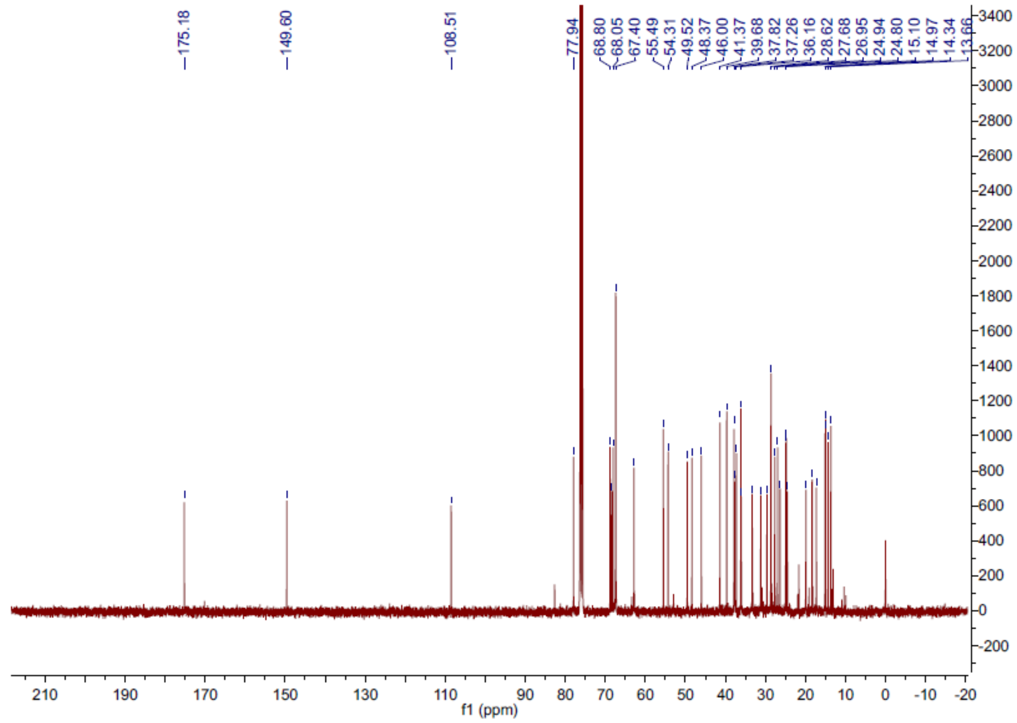

Figure S4. ^{13}C NMR (101 MHz, $CDCl_3$, 298K) of compound BA-G.

Single Mass Analysis
Tolerance = 5.0 PPM / DBE: min = -1.5, max = 50.0
Element prediction: Off
Number of isotope peaks used for i-FIT = 3

Monoisotopic Mass, Even Electron Ions
498 formula(e) evaluated with 1 results within limits (all results (up to 1000) for each mass)
Elements Used:
C: 57-57 H: 0-90 N: 0-12 O: 0-10 S: 1-2 Fe: 0-1
9 216 (1.214)
1: TOF MS ES+

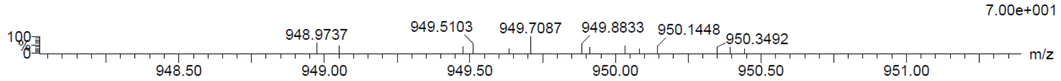

Minimum:				-1.5				
Maximum:		5.0	5.0	50.0				
Mass	Calc. Mass	mDa	PPM	DBE	i-FIT	Norm	Conf(%)	Formula
949.5103	949.5103	0.0	0.0	17.5	76.8	n/a	n/a	C57 H81 O6 S Fe

Figure S5. HRMS of BA-G.

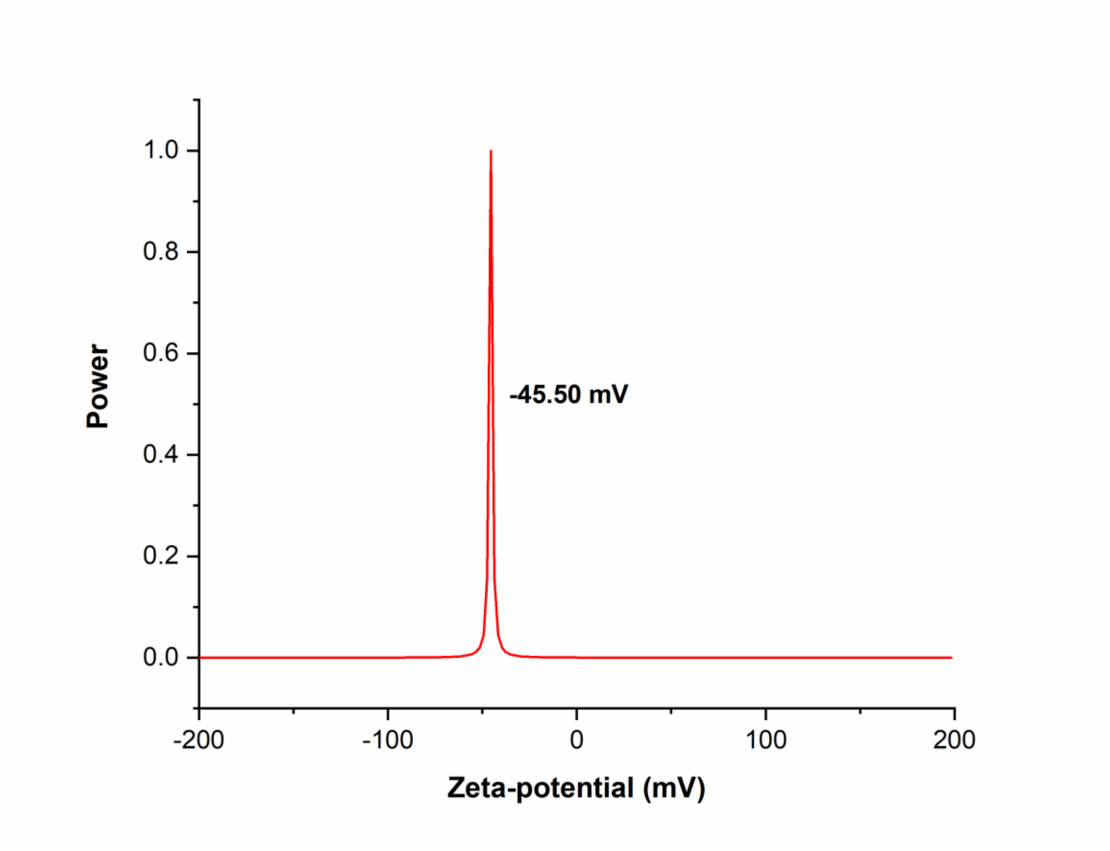

Figure S6. Zeta-potential of BNPs nanoparticles.

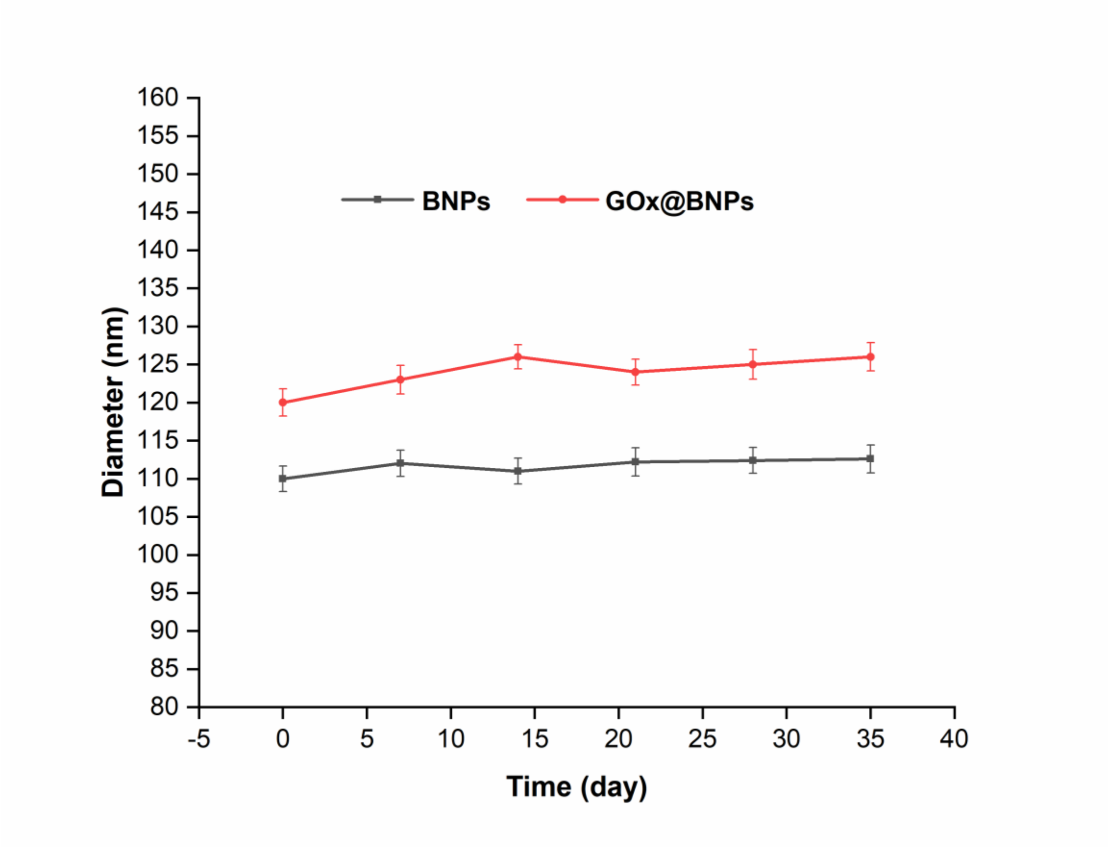

Figure S7. Stability of the BNPs and GOx@BNPs. Time-dependent size changes of BA-NPs and GOx-loaded nanoparticles GOx@NPs were determined by DLS.
